# Prevalence and associated risk factors of transfusion transmissible infections among blood donors at Regional Blood Transfusion Center Nakuru and Tenwek Mission Hospital, Kenya

**DOI:** 10.11604/pamj.2019.34.31.17885

**Published:** 2019-09-16

**Authors:** Grace Bartonjo, Joseph Oundo, Zipporah Ng’ang’a

**Affiliations:** 1 National Public Health Laboratory Services (NPHLS), Ministry of Health, Nairobi, Kenya; 2 Field Epidemiology and Laboratory Training Program, Kenya; 3 Institute of Tropical Medicine and Infectious Diseases, Jomo Kenyatta University of Agriculture and Technology, Juja, Kenya; 4 United States Army Medical Research Unit, Nairobi, Kenya

**Keywords:** TTIs, prevalence, blood donors, risk factors, HIV, hepatitis C virus (HCV), hepatitis B virus (HBV), syphilis, malaria, Kenya

## Abstract

**Introduction:**

Unsafe transfusion practices can put millions of people at risk of Transfusion Transmissible Infections (TTIs). In Kenya the current blood transfusion scheme involves screening of blood for HIV, Hepatitis B virus (HBV), Hepatitis C virus (HCV) and syphilis. Malaria is also a blood-borne disease which is not currently screened for. In Kenya blood donor selection criteria were reviewed in 2009. Regular review of effectiveness of donor selection criteria can help reduce TTIs prevalence amongst donors and thus make the blood supply safer.

**Methods:**

A cross sectional study was conducted between November 2011 to January 2012 among 594 blood donors in the Regional Blood Transfusion Center Nakuru and Tenwek Mission Hospital. Socio-demographic characteristics and associated risk factors were collected using a standard blood transfusion service questionnaire. Donors were obtained through systematic sampling. Each donor sample was screened, for HIV-1 and HIV-2, HBV, HCV, syphilis and malaria parasites.

**Results:**

The overall prevalence of TTIs was 14.1%, which ranged from 0.7% for malaria to 5.6% for HBsAg. Blood donors who were married (P=0.0057), had non-formal or just primary education (P=0.0262), had multiple sexual partners (P=0.0144) and in informal occupation (P=0.0176) were at higher risk of HIV positivity. History of blood transfusion/blood products (P=0.0055), being married (P=0.0053) were high risk factors associated with positive syphilis. Being male (P=0.0479) was a high risk factor to HBV infection.

**Conclusion:**

The prevalence of TTIs indicates a need to review the questionnaire and apply it strictly for donor selection. The 0.7% prevalence of malaria, poses a serious health risk to non-immune recipients of transfusion. Malaria should be included among mandatory TTI tests in Kenya.

## Introduction

Although timely transfusion saves millions of human lives worldwide each year, unsafe transfusion practices can put millions of people at risk of Transfusion Transmissible Infections (TTIs) [1]. Evaluation of data on the TTIs which include among others; HIV, HBV, HCV, syphilis and malaria, in blood and blood component donors, permits an assessment of the occurrence of infections in the blood donor population and consequently the safety of the donations [2]. It also gives an idea of the epidemiology of these diseases in the community [3]. Transfusion associated infections continue to be a big threat globally [2]. The TTIs can exist asymptomatically in donors, so donors must be screened for high-risk behavior related diseases. In Africa 5-10% of HIV transmission is as a result of contaminated blood transfusions [4]. Infection by HBV and HCV causes serious mortality, morbidity and financial burden thus it is a major global health problem [5]. In Sub-Saharan Africa, 12.5% of patients who receive blood transfusion are at risk of post-transfusion hepatitis [6]. Syphilis is less readily transmitted by blood and the prevalence is low in most studies reported [2]. In Africa a child dies every 45 seconds of malaria, the disease accounts for 20% of all childhood deaths [3]. Studies conducted in Benin revealed that there is a presence of plasmodium falciparum in 30.2 and 33.5% of blood donors respectively [7].

Kenya's need for blood is estimated at between 200,000 and 250,000 units per year. However, with the WHO guidelines estimate of 10-20 units per 1,000 people then the need in Kenya can be estimated at between 380,000-760,000 units annually. Currently, 125,000 units of blood are collected through the National Blood Transfusion Service (NBTS). The deficit is thought to be covered by Family Replacement Donors (FRD) [8]. The FRD account for up to 35 percent of donors in Kenya, despite them being more risky [8]. In this study blood donors were either Volunteer Non-Remunerated Donors (VNRD) or FRD. Nakuru Regional Blood Transfusion Center (RBTC) recruited VNRD from low-risk populations, while in Tenwek Mission Hospital donors were relatives or friends of patients to replace blood used. A standard questionnaire was administered to all consenting donors from the two facilities. Regular review of effectiveness of donor selection criteria can help reduce TTIs prevalence amongst donors and thus make the blood supply safer. Kenya is one of the countries with high prevalence of HIV, HBV, HCV and other blood-borne infections. The population prevalence with HIV (6.0%), HBsAg (8.0%), ant-HCV (2.0%) and syphilis (0.5%) [9]; therefore there is little published data on prevalence of TTIs, socio-demographic characteristics & associated risk factors among blood donors in Kenya.

In Kenya, the current blood transfusion policy recommends screening of blood for HIV, Hepatitis B, Hepatitis C and syphilis. Malaria is also a blood-borne disease which is not screened for. This study included testing for malaria parasites which is not done routinely within the National Blood Transfusion Service (NBTS). While the search for effective therapies and vaccines continues, prevention and control of these blood-borne infections should be the goals of sustained public health efforts. The first step in ensuring blood safety is the selection of low risk blood donors. The NBTS questionnaire was introduced in 2009. The donor selection criterion has not been reviewed since then. Donor selection criteria should be reviewed periodically as disease epidemiology changes [3]. Since introduction, the validity of the questionnaire has not yet been tested. This study will give NBTS very useful information on the strength of the questionnaire in eliminating high risk donors. Many TTIs including HIV and hepatitis have an infectious latent phase when carriers are seemingly healthy. In malaria endemic regions, healthy persons may have low grade parasitaemia that can cause death in non-immune persons. These latency stages pose major challenges to Blood Transfusion Services across the world as they seek to minimize collection of blood from infected persons through appropriate donor selection. To increase the efficacy of donor screening and selection, it is important to understand the risk factors for these infections in different populations [10]. Several studies within Africa, suggest that the African blood donor is mostly young [7], with a mean age of 28.9±8.5 years. This is also the population at highest risk of acquiring HIV and malaria infections. There is need to ensure that the blood the recipients receive is safe from known infections. This study therefore seeks to determine the prevalence, socio-demographic profiles and examine associated risk factors of blood donors at RBTC Nakuru and Tenwek Mission Hospital. The findings will guide in targeting low risk donors and donor selection practices within the Kenya NBTS with the aim of ensuring a safer blood supply in Kenya.

## Methods

**Study design, setting and eligibility:** this was a cross sectional study, whereby data on demographic characteristics and associated risk factors of TTIs was obtained from blood donors at two health facilities. Whereas RBTC Nakuru collects blood from VNRD, Tenwek uses FRD and does not subject them to the standard questionnaire. The inclusion criteria of the study population included: age 16 years and 65 years, body weight greater than 50kg, haemoglobin level greater than 12.5 g/dl and informed consent to participate in the study whereby the exclusion criteria were: age less than 16 years or more than 65 years, body weight less than 50kg. haemoglobin values less than 12.5 g/dl, history of jaundice, sickle cell disease, hypertension or current fever, recent illness or transfusion, high risk sexual behavior and lack of consent.

**Sampling method:** systematic sampling method was conducted between November 2011 and January 2012 among blood donors in the two facilities, who met the facilities criteria for donation. A Sampling frame was created using the blood donor register. The number of blood donations at the two facilities was an average of 1,600 in Nakuru and 120 in Tenwek per month. Due to small workload in Tenwek a proportional allotment was not plausible. An intuitive assignment to Tenwek a sample size of 100 and to Nakuru a sample size of 594-100=494 was done. A random starting point was selected after which every k^th^ person in the population list was selected. The sampling interval was calculated as follows: *K=N/n,* Where *N* was the population size and *n* was the sample size. *k*=3 in Nakuru and 1 in Tenwek. Using this procedure each person in the population had a known and equal probability of selection.

**Data collection:** data, including socio-demographic characteristics such as age, sex, marital status, occupation, residence, level of education and selected risk factors which include history of jaundice, sickle cell disease, hypertension or current fever, recent illness or transfusion, high risk sexual behavior were collected from consenting participants using standard blood transfusion service questionnaire.

**Laboratory test:** the blood of all eligible participants were screened for HIV-1, HIV-2, hepatitis B, hepatitis C virus, syphilis and malaria parasites, per the routine NBTS protocols. Testing was carried out at RBTC Nakuru. Hepatitis B surface antigen (HBsAg) was assayed using Hepanostika hepatitis B surface antigen (Murex Biotech S.A (pty) Ltd, Abbott Murex, Biomerieux, Kyalami Business park, Kyalami boulevard-S.A), and hepatitis C virus antibodies (ant-HCV) using Murex anti-HCV version 4.0 (Murex, Kyalami S.A, Marcy i'etoile, France). Manufacturer's negative and positive controls were included; known negative and positive internal patient samples were used for internal quality control. Presence of antibodies to treponema pallidum were screened using Rapid Plasma Reagin test (RPR) (Omega diagnostics ltd. Omega, hill foots b/v. alva fk 125 dq, Scotland, U.K). HIV-1 and HIV-2 was screened using Vironostika HIV uni-form II Ag/Ab (Murex biotech S.A (pty) Ltd, Abbott Murex, Biomerieux, Kyalami Business Park, Kyalami boulevard-S.A). Manufacturer's serum controls and internal known patient sera controls were included in the test run; these were negative and positive controls. Plasmodium species was screened using SD Bio-line rapid diagnostic test kit (MT Promedt Consulting GmbH, Altenohofstrasse 80 D-66386 St. Ingbert Germany), and confirmed by microscopy using 10% giemsa stained blood films. Internal known negative and positive slides were used for internal quality control.

**Data management and analysis:** prior to any protocol-related procedures being conducted, donors were assigned Unique Identification Numbers (UIN) to allow linkage between information from questionnaires and specimens. All donors information was entered into the study databases (Microsoft excel and Epi-Info files) associated with a UIN in password protected files. Double entry systems for the data were maintained. The questionnaires records were kept in a locked filing cabinet located in a restricted-access room at the research station. The data generated was analyzed using Epi-info 3.5.1 statistical package (CDC, Atlanta, USA). Descriptive analysis was done where measure of central tendency, dispersion and proportions were calculated. Chi square with Yates correction was used to determine any association between socio-demographic characteristic and exposure to risk factors. A p value <0.05 was considered statistically significant. Prevalence odds ratio was used as the measure of association. All variables with a p≤0.1 were subjected to unconditional logistic regression where stepwise, backward and forward elimination logistic regression was used to come up with the final “Best-fit”

**Ethical considerations:** study approval was granted by KEMRI Scientific Steering Committee (SSC), SSC No. 2113, the National Ethical Review Committee and Board of Postgraduates of Jomo Kenyatta University of Agriculture and Technology. Prior to interview, each potential study participant was asked to provide written consent. Participants confidentiality was ensured by coding and omitting information that identifies the participants. Privacy was maintained during interviews.

## Results

**Figure 1:**
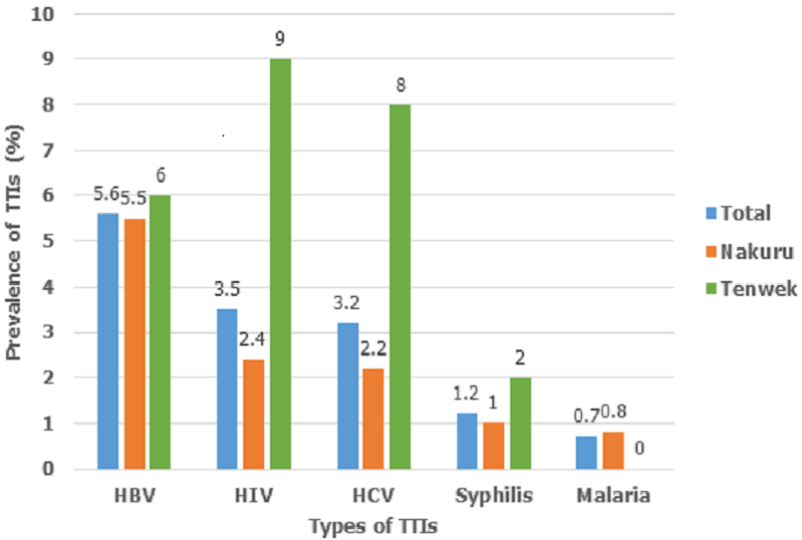
Proportion of Transfusion Transmissible Infections (TTIs) among blood donors

A total of 594 participants were enrolled in the study of which 17% (100) were FRD from Tenwek Mission Hospital and 83% (494) were VNRD from RBTC Nakuru. Of the 594 participants, males constituted 72%. The overall ages of the donors ranged from 16-62 years, median age 20.0 years, RBTC donors had a median age of 19 years, while donors from Tenwek mission hospital had a median age of 25.5 years. Overall 75% of all donors were single, (82% in Nakuru and 40% in Tenwek) (Table 1). The TTIs were more common in males, with HBsAg having the highest prevalence of 6.8%. In Tenwek 22.2% of donors with HBV infection were in age group 36-40 years. HIV sero-prevalence was highest among those with primary as their highest level of education (14.7%) (Table 2). Of the 594 blood donors, the overall prevalence of TTIs was 14.1%. Although Tenwek represented 17% of the sample size, 25% of those with TTIs were from this facility. Individual TTIs were most common among the FRD in Tenwek with a prevalence of 9.0%, 6.0%, 8.0%, 2.0% and 0% of the subjects positive with HIV, HBsAg, ant-HCV, syphilis and malaria. Only four donors had malaria and all of them were from Nakuru (Figure 1).

**Table 1:** Socio-demographic characteristics of blood donors in the Regional blood transfusion Center Nakuru and Tenwek Mission Hospital

	Nakuru & Tenwek	Tenwek Hospital	RBTC Nakuru
Variables	Frequency (%) N=494	Frequency (%) N=100	Frequency (%)N=594
**Sex**			
Male	429(72)	94(94)	94(94)
Female	165(28)	6(6.0)	6(6.0)
**Age-group**			
<20	308(51.9)	19(19.0)	19(19.0)
21-25	126(21.2)	31(31.0)	31(31.0)
26-30	61(10.3)	13(13.0)	13(13.0)
31-35	46(7.7)	20(20.0)	20(20.0)
36-40	30(5.1)	9(9.0)	9(9.0)
40+	23(3.9)	8(8.0)	8(8.0)
**Marital status**			
Married	140(23.6)	60(60.0)	60(60.0)
Single	446(75.1)	40(40.0)	40(40.0)
Divorced	7(1.2)	0(0.0)	0(0.0)
Widowed/wer	1(0.2)	0(0.0)	0(0.0)
**Level of education**			
None	1(0.2)	0(0.0)	0(0.0)
Primary	34(5.7)	18(18.0)	18(18.0)
Secondary	399(67.2)	60(60.0)	60(60.0)
Tertiary	160(26.9)	22(22.0)	22(22.0)
**Occupation**			
Student	367(61.8)	22(22.0)	22(22.0)
Unemployed	24(4.0)	0(0.0)	0(0.0)
Formal	89(15.0)	18(18.0)	18(18.0)
Informal	114(34.2)	60(60.0)	60(60.0)

**Table 2:** Socio-demographic characteristics versus TTIs among blood donors in the Regional Blood Transfusion Center Nakuru and Tenwek Mission Hospital

Variable	HIV Positive / Total donors (%)	HBV Positive/ Total donors (%)	HCV Positive / Total donors (%)	Syphilis Positive/ Total donors (%)	Malaria Positive/Total Donors (%)
**Sex**					
Male	16/429 (3.7)	29/429 (6.8)	17/429 (4.0)	6/429 (1.4)	3/429 (0.7)
Female	5/165 (3.0)	4/165 (2.4)	2/165 (1.2)	1/165 (0.6)	1/165 (0.6)
**Age in years**					
<20	5/308 (1.6)	12/308 (3.9)	9/308 (2.9)	2/308 (0.6)	2/308 (0.6)
21-25	5/126 (4.0)	11/126 (8.7)	6/126 (4.8)	0/126 (0.0)	2/126 (1.6)
26-30	2/61 (3.3)	3/61 (4.9)	6/61 (1.6)	2/61 (3.3)	0/61 (0.0)
31-35	4/46 (8.7)	3/46 (6.5)	1/46 (2.2)	1/46 (2.2)	0/46 (0.0)
36-40	3/30 (10)	3/30 (10)	1/30 (3.3)	1/30 (3.3)	0/30 (0.0)
40+	2/23 (8.7)	1/23 (4.3)	1/23 (4.3)	1/23 (4.3)	0/23 (0.0)
**Marital status**					
Married	12/140 (8.6)	9/140 (6.4)	5/140 (3.6)	5/140 (3.6)	0/140 (0.0)
Single	8/446 (1.8)	24/446 (5.4)	14/446 (3.1)	2/446 (0.4)	4/446 (0.9)
Divorced	1/7 (14.3)	0/7 (0.0)	0/7 (0.0)	0/7 (0.0)	0/7 (0.0)
Widowed/wer	0/1 (0.0)	0/1 (0.0)	0/1 (0.0)	0/1 (0.0)	0/1 (0.0)
**Level of Education**					
None	0/1 (0.0)	0/1 (0.0)	0/1 (0.0)	0/1 (0.0)	0/1 (0.0)
Primary	5/34 (14.7)	2/34 (5.9)	1/34 (2.9)	0/34 (0.0)	0/34 (0.0)
Secondary	14/399 (3.5)	18/0399 (4.5)	13/399 (3.3)	3/399(0.8)	3/399 (0.8)
Tertiary	2/160 (1.3)	13/160 (8.1)	5/160 (3.1)	4/160(2.5)	1/160 (0.6)
**Occupation**					
Student	6/367 (1.6)	20/367 (5.4)	11/367 (3.0)	2/367(0.5)	4/367 (1.1)
Unemployed	2/24 (8.3)	2/24 (8.3)	0/24 (0.0)	0/24 (0.0)	0/24 (0.0)
Formal	8/89 (3.4)	5/89 (5.6)	1/89 (1.1)	5/89 (5.6)	0/89 (0.0)
Informal	10/114(8.8)	6/116 (5.2)	7/114 (6.1)	0/114(0.0)	0/114 (0.0)

Potential factors associated with various TTI statuses among blood donors in the two sites were analyzed. On bivariate analysis, three socio-demographic high risk factors were found to be significantly associated with positive HIV status. Age above 30 years (P-value=0.003), being married (P-value=0.0007), having informal occupation (P-value=0.05), not educated beyond primary level (P-value=0.005) and having multiple sexual activity (P-value=0.02). Low risk factors significantly associated with HIV positive status were: age below 20 years (P-value=0.02), being single (P-value=0.0001) and being a student (P-value=0.02) (Table 3). Being married (P-value=0.009) was a high risk factor significantly associated with syphilis positive status, while being single (P-value=0.01) was a low risk factor among blood donors (Table 4). None of the factors were significantly associated with positive HCV infection.

**Table 3:** Bivariate analysis of donors by HIV status, Kenya 2011

N=594				
Characteristics	HIV positive No (%)	HIV negative No (%)	OR	95% C.I
Married	12(57.1)	128(22.3)	4.64	1.91-11.25
Age >30 years	9 (42.9)	90 (15.7)	4.02	1.65-9.83
Informal occupation	10 (47.6)	106 (18.5)	4.8	2.75-8.37
None/primary education	5 (23.8)	30 (5.2)	5.66	1.94-16.48
Age ≤20 years	5(23.8)	303(52.9)	0.28	0.1-0.77
Single	8(38.1)	438 (76.4)	0.19	0.08-0.47
Student	6 (66.7)	361 (93.8)	0.13	0.03-0.56
Multiple sexual activity	2 (9.5)	5 (0.9)	11.96	2.18-65.6

**Table 4:** Bivariate analysis of donors by syphilis status, Kenya 2011

N=594				
Characteristics	Syphilis positive No. (%)	Syphilis negative No. (%)	OR	95% C.I
Male	6(85.7)	423(72.1)	2.33	0.28-19.47
Married	5(71.4)	135(23.0)	8.37	**1.61-43.63**
Single	2(28.6)	444(75.6)	0.13	**0.02-0.67**
Age ≥30 years	3(42.9)	96(16.4)	3.84	0.85-17.4
Age ≤20 years	2(28.6)	306(52.1)	0.37	0.07-1.91
Blood transfusion/products	1(20.0)	4(0.8)	30.31	2.74-334.86
Had sexual activity with a person background not known	1(14.3)	17(2.9)	5.59	0.64-49.0

All factors that were significant at p≤0.1 on bivariate analysis were subjected to the unconditional logistic regression model using backward stepwise elimination method. The final “best-fit” model contained five factors that were independently associated with positive HIV status: donors who were married (aOR=4.56; P-value=0.0057), having non-formal or just primary education (aOR=9.05; P-value=0.0262), informal occupation (aOR=4.08; P-value=0.0176) and multiple sexual partners (aOR= 189.78; P-value=0.0144). History of previous blood transfusion/blood products (aOR=9727.90; P-value=0.0055) and being married (aOR=12.27; P-value=0.0053) were high risk factors associated with positive syphilis. Being male (aOR=2.92; P-value=0.0479) was a high risk factor for HBV infection (Table 5).

**Table 5:** Final model of factors associated with positive HIV, syphilis & HBV among blood donors in the RBTC Nakuru & Tenwek Mission Hospital, 2011

HIV positive			
Characteristics	Adjusted odds Ratio	95% Confidence Interval	p-value
Married	4.56	1.55-13.40	0.0057
None/primary education	9.05	1.30-63.17	0.0262
Multiple sexual activity	189.78	2.84-12700.67	0.0144
Informal occupation	4.08	1.28-13.04	0.0176
**Syphilis positive**			
Married	12.27	2.11-71.34	0.0053
Blood transfusion/products	9727.90	14.89-6355911.32	0.0055
**HBV positive**			
Male	2.92	1.01-8.43	0.0479

## Discussion

This study revealed that, the majority of blood donors were young males 72%. This may be attributed to deferral of potential female donors due to anaemia, pregnancy, breastfeeding or childbirth which are all criteria for donor exclusion. In this study the ratio of male to female blood donors is similar to other studies [3] and found male: female ratio was 5:1. In the Kenyan survey [9], more men compared to women donated to a blood transfusion service among voluntary and FRDs. A difference in occupation and marital status was noted between the two facilities, whereby in Tenwek Mission Hospital, most donors 60% had informal occupation and 60% were married, while in RBTC Nakuru 70% were students and 82% singles were the predominant group. This is biased by higher predominance of students especially among voluntary donors; most of the recruitments were from secondary school students. These results are in line with those reported by Cunha and his collaborators in Mozambique, who noted that the proportion of volunteer donors (69%) who completed secondary school education was higher than the familial ones [7]. This contrasts with findings from Burkina Faso, which found that more than 31% of their donor population is illiterate or received only primary school education [7].

Overall majority of the participants were aged between 16-20 years with a median age of 20.0 years. Routinely, RBTC targets donors of a younger age, mostly high school and college students, as this group are perceived to be more willing to donate blood and also a low risk group. These findings are similar with a study in Kenya where 59% of voluntary donors were <25 years old [11]. In a review of blood donors in Africa, it was observed that, African blood donors are mostly young. In Kenya, a mean age of 28.9±8.5 years was reported among blood donors; in Burkina Faso, reported a mean age of 28±7.9 years, In Rwanda more than 75% are less than 30 years [7]. These mean ages are 10-15 years less than those observed in European countries. This difference may be explained by the fact that the voluntary donor programmes in Africa tend to be centered on secondary school and university students and (Donor ages tend to relate to the donor type), where the median ages for secondary school, public blood drive and family replacement donors were 18, 25 and 32 years, respectively [7].

The overall prevalence of HIV was more in male than female, this is similar to what was reported in Osogbo, south-west Nigeria [12] in which more male (82%), were infected by HIV than females (18%). The prevalence of HIV in RBTC Nakuru was higher in females 3.1% than in males 2.1%. This HIV sero-positivity among female donors observed in voluntary donors in this study is possibly because, women of all ages are more likely than men to become infected with HIV during unprotected vaginal intercourse which may be attributed to socio-economic, cultural and biological factors which have shown to contribute to the female genders' vulnerability to HIV [6]. A study of transfusion transmissible viral infection among university fresh students in Nigeria [13] reported a prevalence of HIV of female to male to be 1.3% to 0.4% respectively. A study in sub-Saharan Africa shows that there were 12 to 13 HIV-infected women for every 10 infected men [14].

All of the blood donors with syphilis (2.1%) in this study were males and they were all from Tenwek Mission Hospital. Various studies have reported male dominance in syphilis positivity, 96% [15] and 95% [16]. All the donors were males among Sudanese blood donors [17]. Sero-prevalence of HBsAg was more common among blood donors of 36-40 year age group. The higher prevalence rate of HBV among relatively older people in this study indicates that most of these participants may have been infected at earlier stage of their life. Alternatively they may have acquired the infection through sex and possibility also exists of horizontal spread of the infection. The HBV infection usually occurs during infancy and childhood by horizontal transmission among children [7]. The sub-Saharan region is highly endemic with HBsAg carrier rates of 9-20%, whereas 56-98% of the adult population shows evidence of past exposure to HBV infection [7]. Studies in Kenya showed an HBsAg carrier rate of 5-30% [18]. The first peak of HBV infection in Kenya appears to be at early school age, whereas the second peak occurs at puberty and childbearing age [18]. This finding is similar with a previous report in which higher prevalence of HBsAg was observed among the youths in the age groups of 26-36 years and 36-45 years compared to age group of greater than 45 years [6].

In HIV positive donors, the highest infection occurred in the age group 36-40 years and above 40 years of age. The possible reason could be these are age groups which are sexually active and married. It has been shown in this study that being married is significantly associated with positive HIV status (P-value=0.0057). These findings are partly in line with previous results where the most affected were the youth of age group 18-47 years [3, 11]. In this study TTIs are common and a major concern among the blood donors with an overall prevalence of 14.1%. Tenwek Mission Hospital with mainly FRD had the highest prevalence of TTIs (25%), in contrast to 11.9% in Nakuru VNRD, though this difference was statistically not significant. The prevalence difference between the two sites is comparable to other studies. In Kenya on comparison of voluntary and FRD on a population based survey, it was found that there is a prevalence of 7.4% and 2.6% respectively [11]. In Eritrea, the total blood donors positive for serological markers for TTIs was 3.8%, of these, voluntary blood donor positive for TTI markers was 3.5% and 5.1% for FRDs [4]. The high prevalence of TTIs in Tenwek Mission Hospital could be attributed to the fact that most of the times, relatives or friends of the family are brought in to cater for their relatives blood needs. As evidenced by the findings of this study, FRDs present a greater risk than VNRDs. The low prevalence of TTIs in VNRDs in this study could be attributed to the fact that voluntary donors are recruited from low-risk populations, coupled with well-trained blood donor staffs at the RBTC who practice effective donor education, stringent donor selection, and no pressure associated with self-deferral as is likely to happen with FRDs. This blood donor recruitment strategy is advocated for by the World Health Organization to improve overall blood safety [19].

In this study the prevalence of all the individual TTIs markers, except malaria was higher among the FRDs than VNRD. This difference was statistically significant for HIV (P=0.0025) and HCV (P=0.0051). It is conceivable that a person in need of money is more likely to conceal his/her true state of health. Monetary motivation of donors might be highly appealing to people who live in desperate financial need. It has been observed that FRDs are more likely to transmit TTIs than are voluntary donors [12]. These findings are similar to a study in India [20] which showed that replacement donors have higher sero-activity rates than voluntary donors due to a number of factors including high risk behaviour and paid donors posing as relatives. The overall prevalence of TTI agents, HBsAg 5.6%, HIV 3.5%, ant-HCV 3.2% and syphilis 1.2% respectively are higher than previously found in Kenya where they reported prevalence of HBV, HIV, ant-HCV and Syphilis of 3.2%, 1.3%, 1% and 0.5% respectively, among blood donors of TTIs [21] and in Kerala where the prevalence were HBsAg 1.3%, HIV 0.2%, ant-HCV 1.4%, and syphilis 0.2% [22]. However these findings are lower than the prevalence of HBsAg 18.6, ant-HCV 6.0% but differ with regards to HIV (3.1%) for which higher prevalence was observed in the current study, but similar (1.1%) with syphilis in the study done in Osogbo, south-west Nigeria [12].

Overall, the commonest TTI was HBsAg with 5.6% positivity rate. The possible reason for the high rate of HBV is a high prevalence in the general population arising from high infectivity potential of the virus, low immunization status and bloodletting exercises to treat different diseases [22]. This data is similar with earlier reports that Kenya is a high endemicity area for HBV [18]. Studies in Kenya showed HBsAg carrier rates of 5-30% [18]. Hepatitis B virus therefore is highly contagious and relatively easy to be transmitted from one infected individual to another. This is because HBV virus is present in all body fluids and secretions, including blood, saliva, semen, sweat, breast milk, tears and urine, and therefore, virus is transmitted through various routes, apparently depending on the incidence of the disease in the region [18]. Previous study has reported that prevalence of an infection among the donors reflects the disease burden in the society [23]. This figure (5.6%) prevalence of HBsAg in the current study is higher than 4% in Kenyan donors [24], 4.3% in Egyptian donors [25]. It is lower than 14% among blood donors in Zimbabwe, southern Africa [13]. In this study 1.2% of study participants had *Treponema Pallidum.* The reasons for the relatively lower rate of sero-prevalence, is conceivable that syphilis is less often transmitted by blood. The prevalence is low in most studies reported [5], 1.2% of syphilis among Kenyan donors [24], 1.2% found in northern-eastern, Nigeria [15]. However it is lower than 3.96% reported in Burkina Faso [26], but is higher than 0.05% among donors in south India. The low prevalence of syphilis in this study might be through increased awareness of the disease and prompt treatment which is cheap and effective. Antenatal screening and treatment for syphilis might also have contributed to low prevalence of syphilis.

There is scant information on malaria among blood donors. Malaria screening test has not been incorporated as one of the routine tests in NBTS panel of testing. In this study a prevalence of malaria of 0.7% was found. This study was conducted in a low-malaria prevalence region which gets malaria only in the rainy seasons from April to October (non-endemic region). This can be anticipated that, during the high season the prevalence can be higher. This is almost in accordance with previous study [27] which explains the low prevalence of malaria parasites (1%) among blood donors was due to the sampling period (December to February) as explained by the seasonal changes in mosquito density. The prevalence of 0.7% of Transfusion Transmissible Malaria (TTM) in this study contrasts with 46.5% for *Plasmodium falciparum* parasites among voluntary blood donors in Nigeria. In the Nigerian study, donors with history of past infections had higher infection rate of 54.5% for malaria than those without past history of infection 46.0%. Donors with previous history of blood transfusion had higher infection rate of 60.0% for malaria than those without such history 46.2% [3]. Studies conducted in Benin revealed the presence of *Plasmodium falciparum* parasites in 30.2 and 33.5% of blood donors respectively [7]. Being married (P-value=0.0057) was a high risk factor for HIV positive status. This is not surprising considering that HIV is a sexually transmitted infection. In many countries there is risk of HIV infection within marriage. In a study in India, 90% of women being treated for STI had only one lifetime partner, and 14% were HIV-positive. In Kisumu, Kenya and Ndola, Zambia, adolescent married girls' aged 15-19 years were found to have higher prevalence of HIV infection than non-married sexually active girls of the same age, demonstrating that marriage can increase risk of HIV infection [28]. Non-formal or primary education status was a high risk factor to being positive HIV (P-value=0.0262). The sero-prevalence of HIV in this study was found to decrease with increasing level of education among VNRD. This might be attributed to the fact that as the level of education increases there is high probability of being aware of preventive measures against HIV infection. Additionally, it is likely that those with high education understand criteria for self-deferral better. Some studies suggest that better educational attainment may correlate with a lower risk of infection among blood donors [7].

Multiple sexual activity was statistically associated with positive HIV (P-value=0.0144). Being a sexually transmitted disease, it is not surprising that increased exposure to sexual activity is associated with increased HIV prevalence. The key mode of acquiring HIV in Africa is sexual activity, multiple partners being one of the main risk factor [29]. Informal occupation (P-value=0.0176) was identified to be independently associated with positive HIV status. These informal workers constitute of business people and farmers which is a major portion of the general population in this region. Farming is a predominant occupation in Tenwek while business is, in Nakuru. Low risk factors significantly associated with HIV positive status were; age below 20 years, being single, highest educational status of secondary or tertiary education and being a student, though these associations were not statistically significant during multivariate analysis. This might be attributed through the pre-donation counseling and education, also ongoing educational programmes targeting mostly the youth. Studies worldwide indicate that the volunteers, who are composed mostly of students, produce the safer blood supply. In Kenya, school students are the main donor group as they are perceived by NBTS to be the low risky group and more willing to donate blood [21].

History of blood transfusion/blood products (P-value=0.0055), was identified to be high risk factor independently associated with positive syphilis. The possible reason for this association is not clear and further evaluation is needed. Being married (P-value=0.0053) was a high risk factor statistically associated with positive syphilis. The opinion may be, since syphilis is being primarily transmitted by sexual route, the transfusion risk of syphilis is closely related to the sexual behaviours. The presence of syphilis points towards donors' indulgence in high-risk behaviours [12] and consequently higher risk of exposure to infections such as HIV [17]. Being male (P-value=0.0479), was identified to be independently associated with positive HBsAg. This association might be attributed by cultural practices which could expose to HBV infection like circumcision using non-sterile equipment. In a study conducted in Nigeria in 2009, a positive association was noted between sex and hepatitis B (χ^2^=9.589, P < 0.002) [13]. None of the factors were significantly associated with positive HBsAg and ant-HCV at an alpha level of significance of 5%. The possible reason could be the questionnaire was not able to pick for the risk factors. This might be due to lack of knowledge and awareness of hepatitis by the blood donors. In a study by olokoba in 2009, none of the risk factors examined were significantly associated with the carriage of HBV infection [30].

**Limitations of the study**: responses from donors could not be verified and no effort was made to do so. None of the donors in Tenwek responded positively to any of the risk factors. It is likely that risky behaviours of these blood donors were purposely denied so as not to lose face amongst family and friends who had approached the donor on behalf of a sick relative requiring transfusion. Such pressure would not be there for VNRDs. It is important to point out that the results obtained in this study do not reflect the prevalence of markers of TTIs in the unselected general population because blood donors are a pre-selected group and all of them are within the sexually active age group.

## Conclusion

The prevalence of TTIs identified among apparently healthy blood donors is significant and a public health problem. The ministry of health and other stake holders should ensure that low risk donor populations as identified in this study are targeted for donations. Interventions aiming at changing high-risk behaviours in the general population to enhance not only safety of the donated blood products but also of the donors themselves should be strengthened. In addition NBTS need to include malaria in the TTI screening panel. The NBTS questionnaire should stringently be applied to defer respondents with high risk behavior and disease conditions.

### What is known about this topic

- The current blood transfusion policy recommends screening of blood for HIV, Hepatitis B, Hepatitis C and syphilis among blood donors;
- Hepatitis B is the most common TTI among blood donors.

### What this study adds

- The profile of the low risk blood donor is a young, single aged between 16 to 20 years, student with secondary education;
- This study included testing for malaria parasites which is not done routinely within NBTS.

## Competing interests

The authors declare no competing interests.
